# Predictive Validity of Hospital-Associated Complications of Older People Identified Using Diagnosis Procedure Combination Data From an Acute Care Hospital in Japan: Observational Study

**DOI:** 10.2196/68267

**Published:** 2025-02-06

**Authors:** Seigo Mitsutake, Tatsuro Ishizaki, Shohei Yano, Takumi Hirata, Kae Ito, Ko Furuta, Yoshitomo Shimazaki, Hideki Ito, Alison Mudge, Kenji Toba

**Affiliations:** 1 Human Care Research Team Tokyo Metropolitan Institute for Geriatrics and Gerontology Tokyo Japan; 2 Australian Institute of Health Innovation Macquarie University Sydney Australia; 3 The Salvation Army Booth Memorial Hospital Tokyo Japan; 4 Department of Psychiatry Tokyo Metropolitan Institute for Geriatrics and Gerontology Tokyo Japan; 5 Department of Pharmacy Tokyo Metropolitan Institute for Geriatrics and Gerontology Tokyo Japan; 6 Tokyo Metropolitan Institute for Geriatrics and Gerontology Tokyo Japan; 7 Department of Internal Medicine and Aged Care Royal Brisbane and Women’s Hospital Brisbane Australia; 8 Centre of Health Services Research The University of Queensland Brisbane Australia

**Keywords:** delirium, functional decline, Japan, older adult, routinely collected health data, elder, hospital complication, HAC-OP, incontinence, pressure injury, inpatient care, diagnosis procedure combination, predictive validity, hospital length of stay, administrative data, acute care, index hospitalization, diagnostic code, linear regression, logistic regression, long-term care, retrospective cohort, observational study, patient care, gerontology, hospital care, patient complication

## Abstract

**Background:**

A composite outcome of hospital-associated complications of older people (HAC-OP; comprising functional decline, delirium, incontinence, falls, and pressure injuries) has been proposed as an outcome measure reflecting quality of acute hospital care. Estimating HAC-OP from routinely collected administrative data could facilitate the rapid and standardized evaluation of interventions in the clinical setting, thereby supporting the development, improvement, and wider implementation of effective interventions.

**Objective:**

This study aimed to create a Diagnosis Procedure Combination (DPC) data version of the HAC-OP measure (HAC-OP-DPC) and demonstrate its predictive validity by assessing its associations with hospital length of stay (LOS) and discharge destination.

**Methods:**

This retrospective cohort study acquired DPC data (routinely collected administrative data) from a general acute care hospital in Tokyo, Japan. We included data from index hospitalizations for patients aged ≥65 years hospitalized for ≥3 days and discharged between July 2016 and March 2021. HAC-OP-DPC were identified using diagnostic codes for functional decline, incontinence, delirium, pressure injury, and falls occurring during the index hospitalization. Generalized linear regression models were used to examine the associations between HAC-OP-DPC and LOS, and logistic regression models were used to examine the associations between HAC-OP-DPC and discharge to other hospitals and long-term care facilities (LTCFs).

**Results:**

Among 15,278 patients, 3610 (23.6%) patients had coding evidence of one or more HAC-OP-DPC (1: 18.8% and ≥2: 4.8%). Using “no HAC-OP-DPC” as the reference category, the analysis showed a significant and graded association with longer LOS (adjusted risk ratio for patients with one HAC-OP-DPC 1.29, 95% CI 1.25-1.33; adjusted risk ratio for ≥2 HAC-OP-DPC 1.97, 95% CI 1.87-2.08), discharge to another hospital (adjusted odds ratio [AOR] for one HAC-OP-DPC 2.36, 95% CI 2.10-2.65; AOR for ≥2 HAC-OP-DPC 6.96, 95% CI 5.81-8.35), and discharge to LTCFs (AOR for one HAC-OP-DPC 1.35, 95% CI 1.09-1.67; AOR for ≥2 HAC-OP-DPC 1.68, 95% CI 1.18-2.39). Each individual HAC-OP was also significantly associated with longer LOS and discharge to another hospital, but only delirium was associated with discharge to LTCF.

**Conclusions:**

This study demonstrated the predictive validity of the HAC-OP-DPC measure for longer LOS and discharge to other hospitals and LTCFs. To attain a more robust understanding of these relationships, additional studies are needed to verify our findings in other hospitals and regions. The clinical implementation of HAC-OP-DPC, which is identified using routinely collected administrative data, could support the evaluation of integrated interventions aimed at optimizing inpatient care for older adults.

## Introduction

Hospitalization can impose a heavy physical and psychological burden on older adults, leading to in-hospital complications such as functional decline (the loss of independence in activities of daily living) and delirium [1]. A recent meta-analysis reported that the prevalence of functional decline following acute hospitalization was 30% in older adults aged ≥65 years [2]. Such functional decline is associated with various adverse outcomes, including prolonged hospital length of stay (LOS) and increased risks of hospital readmission and mortality within 30 days after discharge [3,4]. Similarly, delirium is estimated to affect around one quarter of older hospitalized adults [5-7], and is associated with longer stays, higher mortality, and higher health care costs [8,9].

Previous studies have developed and demonstrated the effectiveness of interventions to prevent such complications in hospitalized older adults [10,11]. While these interventions have generally focused on single complications as outcomes, they may also be effective in preventing other complications (eg, delirium prevention programs can also reduce falls) [1,12-16]. This is because in-hospital complications common in older people (often termed “geriatric syndromes”) tend to have overlapping risk factors that can be collectively targeted by complex and multifaceted interventions [1,12-16]. Mudge et al [1] proposed a multicomponent measure of “hospital-associated complications of older people” (HAC-OP) comprising common complications (functional decline, hospital-associated incontinence, hospital-associated delirium, pressure injury, or fall) among older adults admitted to acute care hospitals. In a prospective study using regular structured patient assessments as well as document review, the authors demonstrated a significant graded association between the HAC-OP measure and hospital LOS, facility discharge, and mortality within 6 months after admission. However, collecting this research measure was resource-intensive [1,17], which makes it difficult to replicate in routine practice. Routinely collected administrative data have been examined to assist clinicians, patients, and policy makers in making informed decisions [18,19]. Estimating HAC-OP from routinely collected administrative data could facilitate the rapid and standardized evaluation of interventions in the clinical setting, thereby supporting the development, improvement, and wider implementation of effective interventions.

In Japan, the majority of acute care hospitals use the Diagnosis Procedure Combination (DPC) case-mix patient classification system, which is linked to a lump-sum payment system for inpatients [20]. DPC-compliant hospitals must generate and submit DPC data to the government. These data include administrative claims and discharge abstracts containing patient-level information on diagnoses (recorded using *ICD-10* [*International Statistical Classification of Diseases, Tenth Revision*] codes), treatments, and prescribed drugs. Unlike many other countries, the required data also include activities of daily living (ADL) assessment scores at admission and discharge [20]. Prior studies have used DPC data to assess ADL scores in patients to examine the effects of rehabilitation services and to identify patients at high risk of early readmission in acute care settings [21,22]. Hospital outcomes, such as LOS and readmission within 28 days after discharge from hospital, are published annually for each DPC hospital [23]. However, in-hospital complications are not currently reported or benchmarked as they are in some other countries [24,25]. The systematic and accurate identification of HAC-OP from DPC data would support the evaluation of inpatient care and interventions that target these complications in Japan’s acute care hospitals.

Although the validity of chronic disease diagnoses in DPC data has been reported to be generally high [26], no study has tested the validity of in-hospital complications in DPC data, and complications are often underestimated in administrative datasets [27]. Understanding the current reporting of in-hospital complications and the association with important outcomes such as LOS and discharge destination could inform their use as an efficient and standardized evaluation of system-level interventions. Therefore, this study was conducted to develop a DPC data version of the HAC-OP measure (HAC-OP-DPC), describe the incidence of HAC-OP-DPC in a cohort of older acute care inpatients, and evaluate the predictive validity of this composite measure and its components by assessing associations with LOS and discharge destination.

## Methods

### Study Design and Patients

This retrospective cohort study was conducted using an anonymized DPC database obtained from a large general public acute care hospital (550 beds: 520 beds on general wards and 30 beds on psychiatric wards) in Tokyo, Japan. The DPC data comprised patient-level demographic characteristics, *ICD-10* codes, treatments, and prescribed drugs during all insurance-covered clinical encounters. The study used data from July 2016 to March 2021.

Patients who had been admitted to the study hospital from home or a long-term care facility (LTCF) and discharged during the study period were eligible for inclusion; the first hospitalization episode during the study period was designated the index hospitalization and included in the analysis. We excluded patients aged <65 years, patients discharged within 2 days of admission, patients who died during the index hospitalization, and patients with missing data in the study variables.

### Ethical Considerations

The study protocol was approved by the Ethics Committee of the Tokyo Metropolitan Geriatric Hospital and Institute of Gerontology (approval number R18-20). All procedures followed the ethical guidelines of the Medical and Biological Research Involving Human Subjects established by the Japanese government. Opt-out consent was used because all data were anonymized before being received by the authors.

### Measures

#### HAC-OP-DPC

Based on the original HAC-OP measure [1], we identified the following 5 conditions as HAC-OP-DPC: hospital-associated functional decline, incontinence, delirium, pressure injury, and fall. Each condition was defined using DPC data.

First, hospital-associated functional decline was defined as a decrease in ADL scores for the Barthel Index (BI) components of bathing, dressing, toileting, transfers, mobility, and feeding from hospital admission to discharge. In the BI, each component is given a score of 0, 5, 10, or 15 points (maximum scores vary among the components), with higher scores indicating greater independence in that activity [28]. BI was assessed at admission and hospital discharge by bedside nurses. Second, hospital-associated incontinence was defined as a decrease in scores for the BI components of bladder function and bowel function from hospital admission to discharge. Third, hospital-associated delirium was identified based on a recorded diagnosis of delirium (Multimedia Appendix 1) as a postadmission complication and recorded prescriptions of drugs used to manage agitation in delirium (injections of haloperidol or other antipsychotic drugs identified using prescription codes that remained constant throughout the study period). Fourth, hospital-associated pressure injury was identified based on a recorded diagnosis of pressure injury as a postadmission complication and discharge abstract records indicating pressure injury treatment during hospitalization without any similar treatment at admission. Fifth, a hospital-associated fall was identified based on a recorded diagnosis of fall as a postadmission complication. The overall multicomponent HAC-OP-DPC measure was categorized into none, 1, and 2 or more complications based on the count of conditions occurring in each patient.

#### Outcome Measures

The study outcome measures were hospital LOS during the index hospitalization, discharge to other hospitals, and discharge to LTCFs. LOS was calculated as the number of days between the dates of admission and discharge. Discharges to other hospitals, such as rehabilitation hospitals and LTCFs (including special nursing homes, private paid care facilities, and social welfare institutions), were identified using the relevant DPC codes indicating discharge destination.

#### Covariates

Using the subject hospital’s DPC data, we extracted demographic variables of patient sex, age group (65-74, 75-84, and ≥85 years), and annual household income (<¥3.7 million, ≥¥3.7 million, and unknown; ¥1=US $0.0092 in 2016) at the index hospitalization [29]. Income was estimated from the available data about insurance copayments. Insurance copayment rates are the designated rates that patients pay at the point of care in Japan. For patients who have an annual household income below ¥3.7 million (approximately US $34,040; ¥1=US $0.0092), the copayment rates are 10% and 20% for patients aged ≥75 years and 70-74 years, respectively [29]. For patients aged ≥70 years who have an annual household income of ¥3.7 million or higher (¥1=US $0.0092), the copayment rate is 30%. The DPC data did not indicate the copayment rates for patients who received public medical assistance and patients aged 65-69 years. Therefore, income for these cases was categorized as “unknown.”

We calculated variables for disease category, comorbidity, and frailty using *ICD-10* codes. Using previously described methods [30,31], we grouped patients into 12 disease categories based on their recorded primary diagnosis for admission. Next, we determined each patient’s score in the Charlson Comorbidity Index (CCI), which is a weighted index of specific comorbidities that were identified using *ICD-10* codes [32]. CCI scores were divided into 3 categories (0, 1-2, and ≥3). Similarly, we calculated each patient’s Hospital Frailty Risk Score (HFRS), which was developed to identify older adults experiencing frailty with a higher risk of adverse outcomes [33]. The total HFRS ranges from 0 to 99 and was divided into 2 categories (<5 and ≥5). To determine baseline functional dependence and incontinence levels in patients at admission, we analyzed the following 2 variables: dependence in ≥1 ADL items (BI components of bathing, dressing, toileting, transfers, mobility, and feeding) at admission and urinary and fecal incontinence (BI components of bladder function and bowel function) at admission. We dichotomized each of these 2 variables into independent (ie, scoring the maximum score on all components) or dependent (all other patients). We also determined each patient’s location before admission (home or LTCF) and the surgical treatment received during the index hospitalization.

### Statistical Analysis

The chi-square test was used to compare the differences in patient characteristics among the 3 HAC-OP-DPC categories. We generated cross-tabulations to examine the co-occurrences of each complication. Pearson correlation coefficients were calculated to measure the associations between each complication.

The associations between HAC-OP-DPC and LOS were examined using multivariable generalized linear regression models for gamma-distributed data with a log-link function that adjusted for all covariates. Effect sizes for 1 HAC-OP-DPC and ≥2 HAC-OP-DPC were quantified using adjusted risk ratios (ARRs) and their 95% CIs, which indicated the likelihood of having a longer LOS. Next, the associations of HAC-OP-DPC with discharge to other hospitals and discharge to LTCFs were examined using multivariable logistic regression models that adjusted for all covariates. Effect sizes for 1 HAC-OP-DPC and ≥2 HAC-OP-DPC were quantified using adjusted odds ratios (AORs) and their 95% CIs, which indicated the odds of being discharged to another hospital or LTCF. In addition to analyzing the associations between the number of HAC-OP-DPC and the 3 outcomes, we constructed models to examine the associations between individual HAC-OP-DPC components and the outcomes. For the analysis of hospital-associated functional decline, we excluded patients who were already dependent in all the BI components at admission because they could not experience any further functional decline. Similarly, for the analysis of hospital-associated incontinence, we excluded patients who were already dependent in both bladder function and bowel function at admission because they could not experience new-onset incontinence during hospitalization. We also conducted several sensitivity analyses. First, we re-ran the primary analysis for each of the 3 outcomes, excluding patients who could not experience functional decline (fully dependent on admission) or hospital-associated incontinence (incontinent on admission). Second, we recalculated the HAC-OP-DPC composite outcome excluding cases of delirium that were identified only by drug prescribing as we recognized that antipsychotic drugs might be prescribed for other indications (eg, behavioral and psychological symptoms of dementia, psychosis). All analyses were conducted using SPSS (version 28.0; IBM Corp), and the cross-tabulations were created using R (version 4.3.2; R project for Statistical Computing). *P* values (2-tailed) below .05 were considered statistically significant.

## Results

### Study Participant Selection

**Figure 1:**
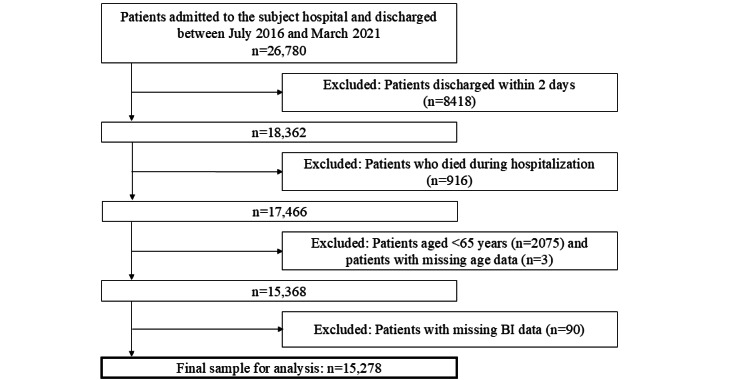
Flowchart of patient selection. BI: Barthel Index.

We first identified 26,780 candidate patients who were admitted to the subject hospital and discharged to home, another hospital, or an LTCF during the study period (Figure 1). After applying the exclusion criteria, the final sample for analysis consisted of 15,278 patients aged ≥65 years. Multimedia Appendix 2 shows the characteristics among 833 patients ≥65 years who died during hospitalization and without missing data at admission. These excluded participants were aged ≥85 years, had ≥2 CCI, were more likely to have ADL dependency and incontinence, and were more likely to have cancer, pneumonia, or heart failure.

### Characteristics of Study Patients

Their mean age was 81.2 (SD 7.9) years, and women accounted for 55.4% of all patients (Table 1). There were 2877 (18.8%) patients who experienced 1 HAC-OP-DPC and 733 (4.8%) patients who experienced ≥2 HAC-OP-DPC during the index hospitalization.

**Table 1:** Patient characteristics according to the number of HAC-OP-DPC^a^.

Characteristics		Total (N=15,278)			HAC-OP-DPC										
					None (n=11,668)			1% (n=2877)			≥2% (n=733)			*P* value	
		N	%	n		%	n		%	n		%			
**Sex**															.16
	Men	6815	44.6	5183		44.4	1280		44.5	352		48.0			
	Women	8463	55.4	6485		55.6	1597		55.5	381		52.0			
**Age (years)**															<.001
	65-74	3353	21.9	2771		23.7	508		17.7	74		10.1			
	75-84	6396	41.9	5009		42.9	1117		38.8	270		36.8			
	≥85	5529	36.2	3888		33.3	1252		43.5	389		53.1			
**Annual** **household income** **(** **¥; ¥** **1=US $0.0092)**															<.001
	<3.7 million	11,600	75.9	8759		75.1	2230		77.5	611		83.4			
	≥3.7 million	1570	10.3	1195		10.2	308		10.7	67		9.1			
	Unknown	2108	13.8	1714		14.7	339		11.8	55		7.5			
**Primary diagnosis for admission**															<.001
	Musculoskeletal diseases	1127	7.4	890		7.6	203		7.1	34		4.6			
	Coronary heart disease	338	2.2	288		2.5	47		1.6	3		0.4			
	Congestive heart failure	689	4.5	505		4.3	133		4.6	51		7.0			
	Cerebrovascular disease	1039	6.8	874		7.5	133		4.6	32		4.4			
	Pneumonia or acute bronchitis	1323	8.7	974		8.3	274		9.5	75		10.2			
	Fracture	576	3.8	407		3.5	137		4.8	32		4.4			
	Metabolic diseases	972	6.4	735		6.3	186		6.5	51		7.0			
	Renal diseases	879	5.8	646		5.5	190		6.6	43		5.9			
	Neurological diseases	631	4.1	510		4.4	100		3.5	21		2.9			
	Gastrointestinal diseases	1694	11.1	1272		10.9	352		12.2	70		9.5			
	Cancer	1867	12.2	1344		11.5	400		13.9	123		16.8			
	Other	4143	27.1	3223		27.6	722		25.1	198		27.0			
**CCI^b^**															<.001
	0	9041	59.2	7116		61.0	1563		54.3	362		49.4			
	1-2	4852	31.8	3578		30.7	1000		34.8	274		37.4			
	≥3	1385	9.1	974		8.3	314		10.9	97		13.2			
**HFRS^c^**															<.001
	<5	13,278	86.9	10,324		88.5	2371		82.4	583		79.5			
	≥5	2000	13.1	1344		11.5	506		17.6	150		20.5			
Dependence in ≥1 ADL^d^ items at admission		12,658	82.9	9764		83.7	2239		77.8	655		89.4	<.001		
Urinary and fecal incontinence at admission		5158	33.8	3932		33.7	1038		36.1	188		25.6	<.001		
**Location before admission**															<.001
	Home	14,186	92.9	10,875		93.2	2642		91.8	669		91.3			
	LTCF^e^	1092	7.1	793		6.8	235		8.2	64		8.7			
Surgical treatment		3337	21.8	2444		20.9	732		25.4	161		22.0	<.001		

^a^HAC-OP-DPC: hospital-associated complications of older people-Diagnosis Procedure Combination data version.

^b^CCI: Charlson Comorbidity Index.

^c^HFRS: Hospital Frailty Risk Score.

^d^ADL: activities of daily living.

^e^LTCF: long-term care facility.

### HAC-OP-DPC Among This Study’s Patients

**Figure 2:**
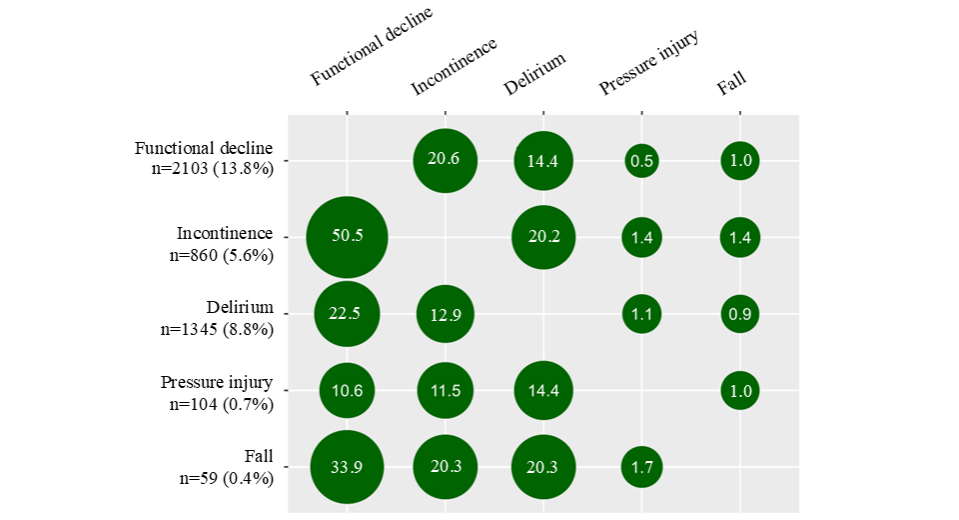
Co-occurrence of each HAC-OP-DPC among older patients aged ≥65 years (N=15,278). Values show the percentages of patients in each row who also had the column condition during hospitalization. HAC-OP-DPC: hospital-associated complications of older people-Diagnosis Procedure Combination data version.

The most common complication was functional decline (n=2103, 13.8%), followed by delirium (n=1345, 8.8%: recorded diagnosis of delirium (n=59); recorded prescriptions of drugs for delirium, n=1286), new incontinence (n=860, 5.6%), pressure injury (n=104, 0.7%), and fall (n=59, 0.4%). Figure 2 demonstrates the patterns of co-occurrence amongst HAC-OP-DPC. For example, among patients with functional decline, 20.6% also experienced incontinence during hospitalization, while 50.5% of patients with incontinence, 33.9% of patients with falls, and 22.5% of patients with delirium also experienced functional decline during hospitalization. While there were statistically significant correlations between most complications, the strength of these correlations was weak (maximum correlation coefficient of 0.26; Table 2). As shown in Table 1, older age, annual household income, multimorbidity, frailty, baseline functional impairment and incontinence, and living in LTCF were all significantly associated with HAC-OP-DPC. Patients with ≥2 HAC-OP-DPC had a significantly greater proportion of patients with congestive heart failure, pneumonia or acute bronchitis, metabolic diseases, and cancer as the principal diagnosis.

**Table 2:** Within-patient correlations between each HAC-OP-DPC^a^ among older patients aged ≥65 years (N=15,278). Values show Pearson correlation coefficients (r) between each HAC-OP-DPC.

	Hospital-associated functional decline	Hospital-associated incontinence	Hospital-associated delirium	Hospital-associated pressure injury	Hospital-associated fall
Hospital-associated functional decline	1.000	0.260^b^	0.079^b^	–0.008	0.036^b^
Hospital-associated incontinence	—	1.000	0.099^b^	0.021^b^	0.040^b^
Hospital-associated delirium	—	—	1.000	0.016^c^	0.025^b^
Hospital-associated pressure injury	—	—	—	1.000	0.008
Hospital-associated fall	—	—	—	—	1.000

^a^HAC-OP-DPC: hospital-associated complications of older people-Diagnosis Procedure Combination data version.

^b^*P*<.01.

^c^*P*<.05.

### Association of HAC-OP-DPC With Outcome Measures

Table 3 demonstrates the significant and graded association observed between HAC-OP-DPC and outcomes. Adjusting for all covariates, those with 1 and ≥2 HAC-OP-DPC were significantly more likely to have a longer LOS during the index hospitalization (ARR 1.29, 95% CI 1.25-1.33, and ARR 1.97, 95% CI 1.87-2.08, respectively) compared to those with no HAC-OP-DPC. They were significantly more likely to be discharged to other hospitals (AOR 2.36, 95% CI 2.10-2.65, and AOR 6.96, 95% CI 5.81-8.35, respectively), and be discharged to LTCFs (AOR 1.35, 95% CI 1.09-1.67, and AOR 1.68, 95% CI 1.18-2.39, respectively). The analyses of individual HAC-OP-DPC showed that patients who experienced hospital-associated functional decline, incontinence, delirium, pressure injury, and falls were significantly more likely to have a longer LOS and be discharged to other hospitals than patients without these complications. However, only delirium was significantly associated in this individual complication analysis with discharge to LCTFs. A sensitivity analysis (n=11,075) that excluded 4203 patients who could neither experience hospital-associated functional decline nor incontinence found that HAC-OP-DPC still had a significant and graded association with longer LOS and discharge destination (Multimedia Appendix 3), similar to the results of the main analysis. Moreover, the associations of the number of HAC-OP-DPC using delirium identified from a recorded diagnosis alone with these outcomes were similar to the results of the main analysis (Multimedia Appendix 4).

**Table 3:** Association of HAC-OP-DPCa with outcome measures in 15,278 participants.

			Participants, n		LOS^b^									Discharge to other hospitals							Discharge to LTCFs^c^				
					Median		IQR		RR^d^ (95% CI)^e^		ARR^f^ (95% CI)^g^		%			OR^h^ (95% CI)^i^		AOR^j^ (95% CI)^k^		%			OR (95% CI)^i^		AOR (95% CI)^k^
**HAC-OP-DPC**																									
	No (Ref^l^)	11,482		14		(7-25)		1.00		1.00		14.4			1.00		1.00		7.3			1.00		1.00	
	1	2,838		18		(9.5-32)		1.28 (1.25-1.32)		1.29 (1.25-1.33)		24.6			1.94 (1.76-2.14)		2.36 (2.10-2.65)		9.7			1.42 (1.24-1.64)		1.35 (1.09-1.67)	
	≥2	724		29		(14-44)		1.88 (1.78-1.99)		1.97 (1.87-2.08)		42.6			4.40 (3.76-5.14)		6.96 (5.81-8.35)		11.1			1.76 (1.38-2.23)		1.68 (1.18-2.39)	
**Hospital-associated functional decline^m^**																									
	No (Ref)	10,124		13		(7-24)		1.00		1.00		10.4			1.00		1.00		4.3			1.00		1.00	
	Yes	2,103		16		(8-31)		1.35 (1.30-1.40)		1.35 (1.30-1.40)		23.6			2.67 (2.37-3.00)		3.56 (3.11-4.09)		7.3			1.77 (1.46-2.14)		1.09 (0.81-1.47)	
**Hospital-associated incontinence^n^**																									
	No (Ref)	10,615		12		(7-22)		1.00		1.00		8.6			1.00		1.00		3.4			1.00		1.00	
	Yes	860		27		(14-42)		1.93 (1.83-2.04)		1.80 (1.71-1.9)		43.3			8.12 (6.98-9.44)		7.33 (6.16-8.73)		13.3			4.39 (3.51-5.49)		1.24 (0.84-1.83)	
**Hospital-associated delirium**																									
	No (Ref)	13,933		14		(8-26)		1.00		1.00		16.1			1.00		1.00		7.6			1.00		1.00	
	Yes	1,345		25		(14-40.5)		1.61 (1.54-1.68)		1.53 (1.47-1.59)		34.1			2.70 (2.39-3.05)		2.23 (1.94-2.56)		12.3			1.71 (1.44-2.04)		1.61 (1.24-2.10)	
**Hospital-associated pressure injury**																									
	No (Ref)	15,174		15		(8-27)		1.00		1.00		17.5			1.00		1.00		7.9			1.00		1.00	
	Yes	104		28		(16.25-42.75)		1.68 (1.45-1.95)		1.43 (1.24-1.64)		48.1			4.37 (2.97-6.43)		2.78 (1.83-4.22)		17.3			2.44 (1.46-4.07)		0.69 (0.31-1.56)	
**Hospital-associated fall**																									
	No (Ref)	15,219		15		(8-27)		1.00		1.00		17.6			1.00		1.00		8.0			1.00		1.00	
	Yes	59		38		(22-50)		2.05 (1.68-2.49)		2.02 (1.68-2.43)		49.2			4.53 (2.72-7.56)		4.65 (2.63-8.23)		5.1			0.62 (0.19-1.98)		0.68 (0.15-3.19)	

^a^HAC-OP-DPC: hospital-associated complications of older people-Diagnosis Procedure Combination data version.

^b^LOS: length of stay.

^c^LTCF: long-term care facility.

^d^RR: risk ratio.

^e^Generalized linear regression analysis.

^f^ARR: adjusted risk ratio.

^g^Generalized linear regression analysis that adjusted for all covariates (sex, age group, annual household income, primary diagnosis for admission, Charlson Comorbidity Index score, Hospital Frailty Risk Score, dependence in ≥1 activities of daily living items at admission, urinary and fecal incontinence at admission, location before admission, and surgical treatment).

^h^OR: odds ratio.

^i^Logistic regression analysis.

^j^AOR: adjusted odds ratio.

^k^Logistic regression analysis that adjusted for all covariates (sex, age group, annual household income, primary diagnosis for admission, Charlson Comorbidity Index score, Hospital Frailty Risk Score, dependence in ≥1 activities of daily living items at admission, urinary and fecal incontinence at admission, location before admission, and surgical treatment).

^l^Ref: reference.

^m^Models for functional decline excluded participants with pre-exiting full dependence (model n=12,227).

^n^Models for hospital-associated incontinence excluded those with pre-existing incontinence (model n=11,475).

## Discussion

### Principal Findings

This retrospective cohort study is the first to develop and apply a tool to assess HAC-OP from routinely collected administrative data and evaluate its predictive validity for hospital outcomes. Our analysis showed that almost one quarter of older inpatients with multiday stays of more than 2 days had a coded HAC-OP, and that having one or more HAC-OP-DPC was associated with longer LOS and discharge to other hospitals and LTCFs. The clinical implementation of the HAC-OP-DPC measure could support comparative analyses of clinical and policy interventions aimed at preventing these complications, thereby contributing to the optimization of acute care for older adults.

Almost 1 in 4 older patients had coding documentation of any HAC-OP-DPC in the present study, which was approximately half that of the incidence of any HAC-OP described in the Australian study [1]. Nonetheless, the incidences of each HAC-OP-DPC were ranked in similar order, with functional decline and delirium being most common. A large contribution to the disparity in incidence is likely to be reliance on DPC data to identify individual complications as HAC-OP-DPC, while the HAC-OP study used repeated patient and clinical record assessments by trained research assistants [1,17]. Although we made efforts to minimize the underestimation of delirium, falls, and pressure injuries by including additional codes and data sources in their identification criteria, it is very likely that the coding data underestimated the incidences of all complications. For example, there were no coded falls without fracture, and only 59 cases of direct recording of a delirium diagnosis. There are recognized gaps in clinician recognition and documentation of hospital complications as well as translation into coding [27]. Understanding and improving the accuracy and usability of HAC-OP-DPC may require correlation with clinical data and comparisons between sites. There are other reasons that our incidence estimates may have been lower than expected. The denominator in our main analysis included 3051 patients who could not experience further functional decline and 3803 patients who could not experience new-onset incontinence, which may have led to an underestimation of these HAC-OP-DPC. Our analyses also excluded patients who died during their inpatient stay, who may have had a higher rate of HAC-OP.

Nonetheless, our study found that the HAC-OP-DPC measure was associated with longer LOS and discharge destination, thereby demonstrating its predictive validity for outcomes in an acute care setting. Furthermore, our analysis found nonoverlapping risk estimates for LOS and discharge to other hospitals between patients with 1 and ≥2 HAC-OP-DPC, showing a significant exposure-outcome effect of a graded nature. These findings were consistent with the results of the original HAC-OP study [1] and suggest that prevention of further HAC-OP is important in those who have already acquired 1 complication. Our observations that HAC-OP-DPC was significantly associated with older age and baseline function are consistent with previous reports [1]. Importantly, our study is the first to demonstrate the strong associations of HAC-OP with higher CCI scores and HFRS, consistent with existing knowledge that comorbidities and frailty are risk factors for the individual complications included in the composite measure [13,16,34-37].

Our analysis also confirmed that the individual HAC-OP-DPC were associated with longer LOS and discharge to other hospitals, which was congruent with previous studies [6,14,35-39]. All HAC-OP-DPC have been individually recognized as important outcomes in older patients [1,6,14,17,36-42]. Although our study showed that there were patterns of co-occurrence and significant correlations among the individual HAC-OP-DPC, these correlations were weak. This suggests that these complications represent relatively distinct conditions and that they can be treated individually [1]. Moreover, a systematic review of composite outcomes in clinical trials proposed that studies should list results for all components of a composite outcome to avoid confusion and bias [43], and an outcome study using the original multicomponent HAC-OP measure demonstrated significant and clinically important improvements in individual outcomes but not in the composite measure [17]. We recommend that future studies should report the effects of interventions not only on the HAC-OP-DPC measure as a composite outcome but also on its individual complications.

### Strengths and Limitations

Strengths of this study include a large, representative dataset with high levels of item completeness (including functional variables), adjustment for important covariates, and use of sensitivity analyses to explore data assumptions. We also recognize several limitations. First, we included additional information to reduce these anticipated underestimates that may have reduced precision; for example, by including drug prescribing of antipsychotics in the delirium diagnosis, we may have included some patients with other indications such as behavioral and psychological symptoms of dementia. However, our sensitivity analysis using a more stringent definition of delirium suggests that this had minimal impact on our overall findings. Second, our study was conducted in a single acute care hospital in Japan, and its findings may not be generalizable to other hospitals, regions, or countries. Nevertheless, the majority of acute care hospitals in Japan have adopted the DPC system [20], and future studies could compare the incidence of HAC-OP-DPC in each acute hospital throughout Japan. Also, although a previous study identified the validity of chronic disease diagnoses in DPC data as being generally high [26], we recognize that the performance of comorbidity scoring methods based on administrative data may vary between health systems [44]. Third, we could not infer a causal relationship between HAC-OP-DPC and LOS because we were unable to identify the date when each complication occurred, and we cannot exclude reverse causality between HAC-OP-DPC and LOS [1]. Fourth, our study did not examine the association between HAC-OP-DPC and mortality, which had been analyzed in the original HAC-OP study [1], because there are no BI scores assigned on discharge for in-hospital deaths to calculate functional decline and incontinence of HAC-OP-DPC, and DPC data lack information on death after discharge. Future studies could link DPC data with mortality data to examine the associations between HAC-OP-DPC and mortality. Finally, our database lacked information on potentially important confounders, such as residential status (eg, living alone or with others), the presence of caregivers, and disease severity that could be included in future studies.

### Conclusions

This study showed that almost one quarter of older acute care inpatients in a Japanese hospital have coding indicating a HAC-OP and demonstrated the predictive validity of the HAC-OP-DPC measure for longer LOS and discharge to other hospitals and LTCFs. To attain a more robust understanding of these relationships, additional studies are needed to verify our findings in other hospitals and regions. The clinical implementation of HAC-OP-DPC, which are identified using routinely collected administrative data, could support the efficient evaluation of integrated interventions aimed at optimizing inpatient care for older adults.

## Appendix

Multimedia Appendix 1 ICD-10 (International Statistical Classification of Diseases, Tenth Revision) codes for identifying hospital-associated complications of older people-Diagnosis Procedure Combination data version.

Multimedia Appendix 2 Characteristics among patients who died during hospitalization (n=833).

Multimedia Appendix 3 Association of HAC-OP-DPC (hospital-associated complications of older people-Diagnosis Procedure Combination data version) with the outcome measures after excluding patients who could neither experience hospital-associated functional decline nor incontinence (n=11,075).

Multimedia Appendix 4 Association of HAC-OP-DPC (hospital-associated complications of older people-Diagnosis Procedure Combination data version) with the outcome measures after excluding patients who could neither experience hospital-associated functional decline nor incontinence (N=15,278).

## Abbreviations

ADL: activities of daily living
AOR: adjusted odds ratio
ARR: adjusted risk ratio
BI: Barthel Index
CCI: Charlson Comorbidity Index
DPC: Diagnosis Procedure Combination
ICD-10: International Statistical Classification of Diseases, Tenth Revision
HAC-OP: hospital-associated complications of older people
HAC-OP-DPC: hospital-associated complications of older people-Diagnosis Procedure Combination data version
HFRS: Hospital Frailty Risk Score
LOS: length of stay
LTCF: long-term care facility
