# Supplementary material for: Predictive Validity of Hospital-Associated Complications of Older People Identified Using Diagnosis Procedure Combination Data From an Acute Care Hospital in Japan: Observational Study
Source: JMIR Aging. 2025 Feb 6;8:e68267. doi: 10.2196/68267 (PMC11843060; doi:10.2196/68267)
Supplement: Multimedia Appendix 1 [file aging_v8i1e68267_app1.docx]

Table S1. ICD-10 codes for identifying HAC-OP-DPC

| HAC-OP-DPC | ICD-10 codes | Description |
| --- | --- | --- |
| Hospital-associated delirium | F05 | Delirium, not induced by alcohol and other psychoactive substances |
| Hospital-associated pressure injury | L89 | Decubitus ulcer and pressure area |
| Hospital-associated fall or fracture | W01 | Fall on same level from slipping, tripping and stumbling |
|  | W05 | Fall involving wheelchair |
|  | W06 | Fall involving bed |
|  | W07 | Fall involving chair |
|  | W08 | Fall involving other furniture |
|  | W10 | Fall on and from stairs and steps |
|  | W18 | Other fall on same level |
|  | W19 | Unspecified fall |
|  | S02 | Fracture of skull and facial bones |
|  | S12 | Fracture of neck |
|  | S22 | Fracture of ribs, sternum, and thoracic spine |
|  | S32 | Fracture of lumbar spine and pelvis |
|  | S42 | Fracture of shoulder and upper arm |
|  | S52 | Fracture of forearm |
|  | S62 | Fracture of wrist and hand level |
|  | S72 | Fracture of femur |
|  | S82 | Fracture of lower leg, including ankle |
|  | S92 | Fracture of foot, except ankle |

Abbreviations: HAC-OP-DPC, hospital-associated complications of older people–Diagnosis Procedure Combination data version; ICD-10, International Classification of Diseases, 10th Revision
