# Supplementary material for: Predictive Validity of Hospital-Associated Complications of Older People Identified Using Diagnosis Procedure Combination Data From an Acute Care Hospital in Japan: Observational Study
Source: JMIR Aging. 2025 Feb 6;8:e68267. doi: 10.2196/68267 (PMC11843060; doi:10.2196/68267)
Supplement: Multimedia Appendix 2 [file aging_v8i1e68267_app2.docx]

| Table S2. Characteristics among patients who died during hospitalization (n=833) | | | |
| --- | --- | --- | --- |
| Characteristics | | Total n=883, % | |
|  |  |  |  |
|  |  | n | % |
| Sex | Men | 426 | 51.1 |
|  | Women | 407 | 48.9 |
| Age, years | 65–74 | 146 | 17.5 |
|  | 75–84 | 313 | 37.6 |
|  | ≥85 | 374 | 44.9 |
| Annual household income | <¥3.7 million | 622 | 74.7 |
|  | ≥¥3.7 million | 97 | 11.6 |
|  | Unknown | 114 | 13.7 |
| Primary diagnosis for admission | Musculoskeletal diseases | 5 | 0.6 |
|  | Coronary heart disease | 11 | 1.3 |
|  | Congestive heart failure | 61 | 7.3 |
|  | Cerebrovascular disease | 39 | 4.7 |
|  | Pneumonia/acute bronchitis | 91 | 10.9 |
|  | Fracture | 5 | 0.6 |
|  | Metabolic diseases | 28 | 3.4 |
|  | Renal diseases | 20 | 2.4 |
|  | Neurological diseases | 5 | 0.6 |
|  | Gastrointestinal diseases | 42 | 5.0 |
|  | Cancer | 382 | 45.9 |
|  | Other | 144 | 17.3 |
| CCI^a^ | 0 | 464 | 55.7 |
|  | 1–2 | 226 | 27.1 |
|  | ≥3 | 143 | 17.2 |
| HFRS^b^ | <5 | 747 | 89.7 |
|  | ≥5 | 86 | 10.3 |
| Dependence in ≥1 ADL^c^ items at admission | Yes | 809 | 97.1 |
| Urinary and/or fecal incontinence at admission | Yes | 472 | 56.7 |
| Location before admission | Home | 754 | 90.5 |
|  | LTCF^d^ | 79 | 9.5 |
| Surgical treatment | Yes | 113 | 13.6 |
| ^a^CCI: Charlson Comorbidity Index.  ^b^HFRS: Hospital Frailty Risk Score.  ^c^ADL: activities of daily living.  ^d^LTCF: long-term care facility. | | | |
