# Supplementary material for: Predictive Validity of Hospital-Associated Complications of Older People Identified Using Diagnosis Procedure Combination Data From an Acute Care Hospital in Japan: Observational Study
Source: JMIR Aging. 2025 Feb 6;8:e68267. doi: 10.2196/68267 (PMC11843060; doi:10.2196/68267)
Supplement: Multimedia Appendix 3 [file aging_v8i1e68267_app3.docx]

Table S3. Association of HAC-OP-DPC^a^ with the outcome measures after excluding patients who could neither experience hospital-associated functional decline nor incontinence (n = 11,075)

|  | n | LOS^b^ | | | |  | Discharge to other hospitals | | |  | Discharge to LTCFs^c^ | | |
| --- | --- | --- | --- | --- | --- | --- | --- | --- | --- | --- | --- | --- | --- |
|  |  | Median | (IQR^d^) | RR^e^  (95% CI)^f^ | ARR^g^  (95% CI)^h^ |  | % | OR^i^  (95% CI)^j^ | AOR^k^  (95% CI)^l^ |  | % | OR  (95% CI)^j^ | AOR  (95% CI)^l^ |
| HAC-OP-DPC | | | | | | | | | | | | | |
| No (Ref^m^) | 8,449 | 11 | (6-21) | 1.00 | 1.00 |  | 6.6 | 1.00 | 1.00 |  | 2.8 | 1.00 | 1.00 |
| 1 | 1,987 | 15 | (8-28) | 1.28  (1.25-1.32) | 1.29  (1.25-1.34) |  | 15.8 | 1.94  (1.76-2.14) | 2.36  (2.10-2.65) |  | 5.1 | 1.35  (1.18-1.56) | 1.36  (0.96-1.91) |
| ≥2 | 639 | 28 | (14-44) | 1.88  (1.78-1.99) | 2.06  (1.95-2.19) |  | 40.1 | 4.40  (3.76-5.14) | 6.96  (5.81-8.35) |  | 10.6 | 1.57  (1.23-1.99) | 1.78  (1.13-2.80) |
| ^a^HAC-OP-DPC: hospital-associated complications of older people-Diagnosis Procedure Combination data version.  ^b^LOS: length of stay.  ^c^LTCF: long-term care facility.  ^d^IQR: interquartile range.  ^e^RR: risk ratio.  ^f^Generalized linear regression analysis.  ^g^ARR: adjusted risk ratio.  ^h^Generalized linear regression analysis that adjusted for all covariates (sex, age group, annual household income, primary diagnosis for admission, Charlson Comorbidity Index score, Hospital Frailty Risk Score, dependence in ≥1 activities of daily living items at admission, urinary and fecal incontinence at admission, location before admission, and surgical treatment).  ^i^OR: odds ratio.  ^j^Logistic regression analysis.  ^k^AOR: adjusted odds ratio.  ^l^Logistic regression analysis that adjusted for all covariates (sex, age group, annual household income, primary diagnosis for admission, Charlson Comorbidity Index score, Hospital Frailty Risk Score, dependence in ≥1 activities of daily living items at admission, urinary and fecal incontinence at admission, location before admission, and surgical treatment).  ^m^Ref: reference. | | | | | | | | | | | | | |
