# Supplementary material for: Predictive Validity of Hospital-Associated Complications of Older People Identified Using Diagnosis Procedure Combination Data From an Acute Care Hospital in Japan: Observational Study
Source: JMIR Aging. 2025 Feb 6;8:e68267. doi: 10.2196/68267 (PMC11843060; doi:10.2196/68267)
Supplement: Multimedia Appendix 4 [file aging_v8i1e68267_app4.docx]

Table S4. Association of HAC-OP-DPC^a^ with the outcome measures after excluding patients who could neither experience hospital-associated functional decline nor incontinence (n = 15,278)

|  | n | LOS^b^ | | | |  | Discharge to other hospitals | | |  | Discharge to LTCFs^c^ | | |
| --- | --- | --- | --- | --- | --- | --- | --- | --- | --- | --- | --- | --- | --- |
|  |  | Median | (IQR^d^) | RR^e^  (95% CI)^f^ | ARR^g^  (95% CI)^h^ |  | % | OR^i^  (95% CI) ^j^ | AOR^k^  (95% CI) ^l^ |  | % | OR  (95% CI)^j^ | AOR  (95% CI)^l^ |
| HAC-OP-DPC | | | | | | | | | | | | | |
| No (Ref^m^) | 12,584 | 14 | (8-26) | 1.00 | 1.00 |  | 15.7 | 1.00 | 1.00 |  | 7.8 | 1.00 | 1.00 |
| 1 | 2,226 | 17 | (9-30) | 1.17  (1.13-1.21) | 1.25  (1.21-1.29) |  | 23.0 | 1.60  (1.44-1.79) | 2.57  (2.25-2.92) |  | 8.4 | 1.08  (0.92-1.28) | 1.17  (0.92-1.50) |
| ≥2 | 468 | 30 | (15-47) | 1.91  (1.78-2.04) | 2.02  (1.89-2.15) |  | 47.2 | 4.81  (3.99-5.81) | 8.43  (6.79-10.46) |  | 11.8 | 1.58  (1.19-2.11) | 1.59  (1.04-2.44) |
| ^a^HAC-OP-DPC: hospital-associated complications of older people-Diagnosis Procedure Combination data version.  ^b^LOS: length of stay.  ^c^LTCF: long-term care facility.  ^d^IQR: interquartile range.  ^e^RR: risk ratio.  ^f^Generalized linear regression analysis.  ^g^ARR: adjusted risk ratio.  ^h^Generalized linear regression analysis that adjusted for all covariates (sex, age group, annual household income, primary diagnosis for admission, Charlson Comorbidity Index score, Hospital Frailty Risk Score, dependence in ≥1 activities of daily living items at admission, urinary and fecal incontinence at admission, location before admission, and surgical treatment).  ^i^OR: odds ratio.  ^j^Logistic regression analysis.  ^k^AOR: adjusted odds ratio.  ^l^Logistic regression analysis that adjusted for all covariates (sex, age group, annual household income, primary diagnosis for admission, Charlson Comorbidity Index score, Hospital Frailty Risk Score, dependence in ≥1 activities of daily living items at admission, urinary and fecal incontinence at admission, location before admission, and surgical treatment).  ^m^Ref: reference. | | | | | | | | | | | | | |
